# An emerging zoonotic disease to be concerned about - a review of the nipah virus

**DOI:** 10.1186/s41043-024-00666-5

**Published:** 2024-10-28

**Authors:** Sumit Paliwal, Suneet Shinu, Rubina Saha

**Affiliations:** 1 All India Institute of Medical Sciences, Bibinagar, India; 2 ESIC Medical College & Hospital, Sanathnagar, Hyderabad, India

**Keywords:** Nipah, Virus, Zoonotic, Emerging Disease, Acute respiratory distress syndrome, Outbreak

## Abstract

The Nipah Virus (NiV) was discovered in 1999 in the Sungai Nipah region of Malaysia. It is one of many emerging bat-borne zoonotic viruses that threaten global health security. The Pteropus fruit bats are identified as the natural reservoirs for the virus. NiV belongs to the family of Paramyxoviridae and is mostly present in locations surrounded by water, vegetation, and controlled or protected religious areas. To date, cases of NiV have been identified in Southeast Asian regions, with the highest number of cases in Bangladesh, totalling 305, with a fatality rate of 65%. The highest mortality has been observed in the Indian region, at 73%. NiV is an emerging zoonotic disease that needs to be focused on. The median incubation period is 9.5 days and the clinical features primarily lead to either progressive encephalitis or Acute Respiratory Distress Syndrome.

The diagnosis is conducted in Bio-safety level 3 or level 4 labs through Polymerase chain reaction. Human nasal swabs, throat swabs, urine, blood, and cerebrospinal fluid (CSF) are collected for diagnostic purposes. At present, there is no approved treatment or vaccine for the prevention of the disease. However, research on a vaccine against NiV is being investigated, and a subunit vaccine with NiV-G protein is found to produce potential efficacy. An outbreak in Kerala, a state in India, led to the implementation of an action plan involving lead agencies to combat the sudden surge of the virus. In the current scenario, appropriate preventive strategies are more effective in controlling the virus. However, emphasis should be placed on affordable and efficient diagnostic methods, treatment options, and vaccines to better manage the virus, considering the highest fatality caused by the virus.

## Introduction

Nipah virus (NiV) is a zoonotic pathogen transmitted by bats, causing deadly encephalitis in humans through infected food or direct contact. Pteropus fruit bats are recognized as the natural reservoirs of this virus. The first outbreak of the Nipah virus (NiV) occurred in Malaysia in 1999, after it was discovered in 1998 during the Sungai Nipah outbreak. The virus was initially isolated from a patient in the southwestern Sungai Nipah region. In 2001, the first outbreak of NiV in India emerged in Siliguri, West Bengal, followed by a recurrence in Nadia, West Bengal, in 2007. A more recent outbreak occurred in 2018 in the Kozhikode district of Kerala, a southern state in India. The affected individual was reported to have contracted the virus from fruit bats [1]. In 2024, a 24-year-old student visiting his hometown of Malappuram in the southern Indian state of Kerala became the second person to die from Nipah virus infection in the state this year. This was confirmed by the country’s national laboratory on 15 September. The first person to die from Nipah virus infection this year was a 14-year-old boy who died in June, just days after testing positive for Nipah. Officials in Kerala have been struggling to contain Nipah virus outbreaks. Last September, they closed schools, offices, and public transport in the Kozhikode district in response to the re-emergence of the virus. The current outbreak marks the sixth spillover event since 2018 [2]. 

There are only 20 whole-genome sequences of NiV existing in humans and 10 in bats, despite seeing near annual human outbreaks in Bangladesh. Generating NiV whole-genome sequences from outbreaks has posed significant challenges due to the low viral load in human throat swab and serum specimens. It is important to note that strains from Bangladesh have unequivocally been divided into two distinct clades, which have demonstrably mixed together geographically in Bangladesh over time and space. As these clades have expanded geographically and temporally, no evidence for significant branch and site-specific selection was observed, except for a single site in the Henipavirus L polymerase. However, the Bangladesh clades 1 and 2 are distinguished by mutations that initially occurred in the polymerase, with additional mutations accumulating in the N, G, F, P, and L genes on external branches. Although widespread, NiV does not show significant genetic variation in human forms based on historic geographical and temporal studies. Therefore, future public health measures should address whether NiV within the bat population also exhibits comparable genetic variation, if zoonotic transmission results in a genetic bottleneck and if surveillance techniques are detecting only a subset of NiV [3]. 

Since 2001, there have been reports of smaller outbreaks of Nipah virus in the Indian subcontinent, although they are less severe than those in Malaysia. This suggests that the virus could have been infecting humans sub-clinically or without being detected for many years. In Malaysia, Nipah virus infections have a high case fatality rate, but in South Asia, it is even higher at approximately 70% [4]. Studies in Thailand have identified that pig movement networks can cause widespread disease dissemination [5]. 

The outbreak in Kerala during 2018 was a difficult challenge to overcome. The diagnostic facility was located far away in a virology institute, and even after that, it required the combined efforts of various institutions from both the Centre and State, along with the assistance of NGOs, to control the spread of the disease to a manageable number [6]. 

Nipah virus has been just one of many emerging bat-borne zoonotic viruses that threatens global health security, along with severe acute respiratory syndrome coronavirus (CoV) that emerged in 2003, Ebola virus, and, most recently, the novel coronavirus that emerged in Wuhan, China, in late 2019. This ever-growing list of pathogens have led to increased investments in vaccine development. Approval of effective candidate vaccines would drastically improve our ability to mitigate the impact of the emergence of a more transmissible Nipah virus strain and these efforts should be always commended. Despite commitments to vaccine development, many basic facts about Nipah virus epidemiology, biology, and ecology remain unknown. Till we have a basic grasp on the characteristics of the Nipah virus, our ability to prevent spill-overs and cure the disease will not improve [4]. 

There was a dearth of updated resources regarding the disease which could give an overall idea pertaining to the virus and its epidemiological and clinical characteristics. Such a resource is much needed by frontline health workers, outbreak investigators and clinicians who may encounter the disease in their day-to-day practice as it can resolve confusion with respect to making a diagnosis. Therefore, this review outlines the Epidemiology, Clinical features, Diagnostic evaluation, Treatment and Preventive measures against NiV. Information regarding future developments is also addressed in the relevant sections so that this review can act as a guide towards seeking upcoming studies as and when they arise.

## Epidemiology

### Agent

The Nipah virus is a type of paramyxovirus that belongs to the Henipavirus genus of the Paramyxoviridae family, which is under the Paramyxovirinae subfamily and Mononegavirales order. The Pteropus fruit bat serves as the reservoir host for NiV, and the virus has a half-life of 18 h within the bats’ urine [7]. 

NIV is an enveloped, negative-sense, single-stranded RNA virus with 6 genes, each being a coding sequence when read consecutively from 3’ to 5’ direction and include:

- N – Nucleocapsid.
- P – Phosphoprotein.
- M – Matrix.
- F – Fusion.
- G – Glycoprotein.
- L – Large Polymerase.

Of these, G and F are essential for cell entry into the susceptible hosts. M protein allows viral budding and morphogenesis. L, N and P encode for proteins that coordinate to form the RNA-dependent RNA polymerase or RdRP. P gene also encodes for 3 other proteins other than the P protein, namely, V, W and C proteins which determine virulence.

Genetic Analysis to identify hosts exhibiting higher potential for viral replication using codon usage revealed humans and bats to be the ones the virus showed maximum adaptability towards. The study also helped to choose suitable experimental animals for vaccine and other studies. Of these the African green monkey was the most optimal [8]. 

### Host

**Fig. 1:**
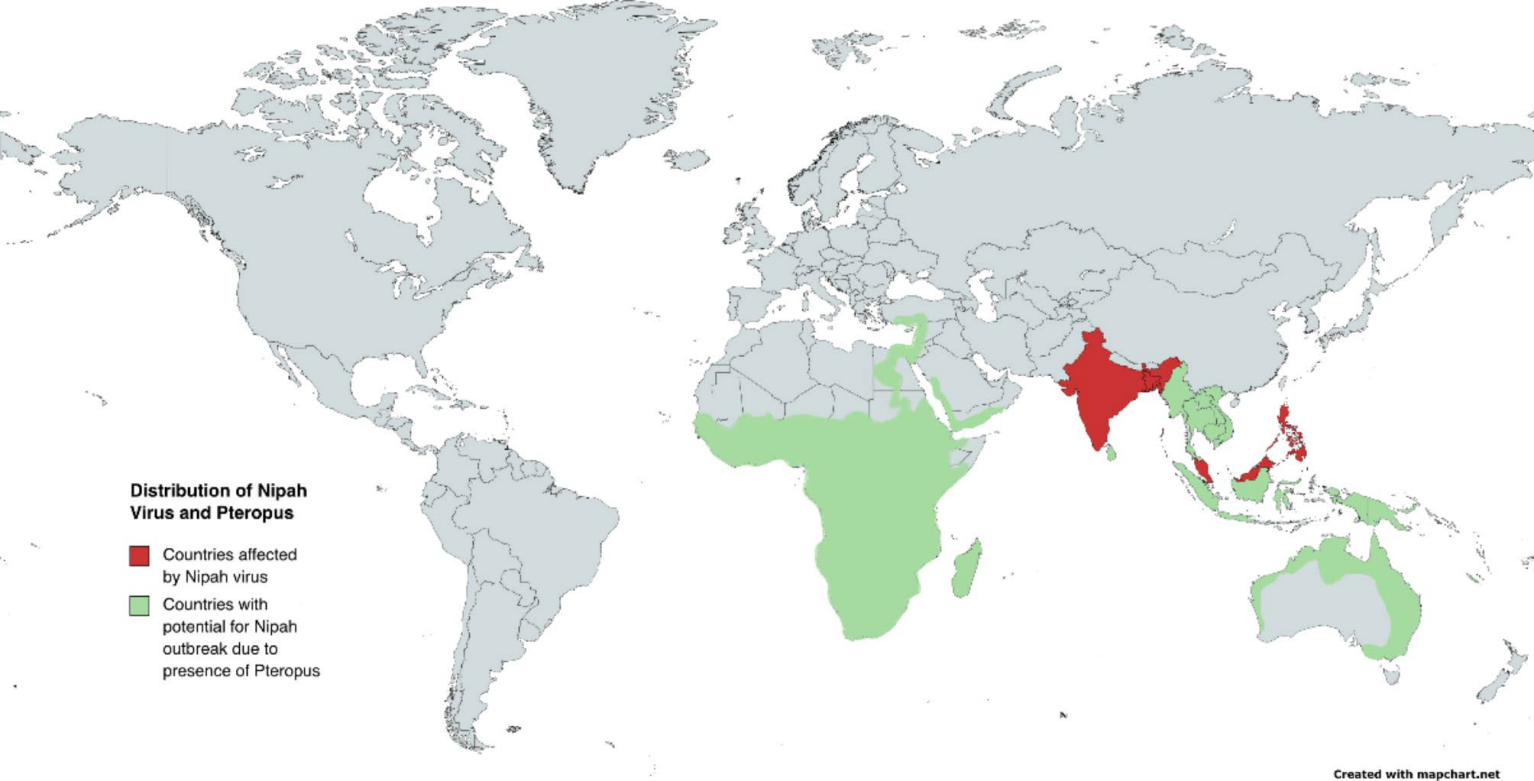
: Distribution of nipah virus affected countries and potential countries under threat of future nipah virus outbreak

The Nipah virus’s natural reservoir is the fruit bats that belong to the Pteropus genus, also known as flying foxes. Pigs, that get infected with the Nipah virus, act as an intermediate host. Furthermore, humans can also become hosts for the Nipah virus [7, 9–11]. Figure 1 depicts the geographical distribution of Nipah virus and *Pteropus spp* [12]. 

### Environment

The destruction and fragmentation of animal habitats can cause increased interaction between wildlife, domestic animals, and humans, thereby increasing the risk of zoonotic disease spread [11]. A study conducted in Thailand aimed to map potential contact zones for bats, pigs, and humans by identifying areas where flying fox colonies are found. The study found that these colonies are typically located in areas surrounded by water, vegetation, and controlled or protected religious sites. In India and Bangladesh, a more recent study used Geographic Information System (GIS) technology to focus on bats carrying the Nipah virus (NiV). The study examined the potential shift in the distribution of the *Pteropus medius* species, using data from 2015 onwards, under various future socioeconomic and environmental scenarios. Additionally, the study predicted an increased risk of NiV transmission events as a result of these shifts. The findings suggested a probable rise in the risk of NiV transmission in these regions due to factors such as population growth and ongoing environmental degradation [11]. The findings of this study were later retracted, but they suggested that it might be crucial to put in place strong public health measures in regions where there is a high risk of the NiV virus being transmitted from *Pteropus medius* bats to humans. Doing so could help to minimize and manage potential NiV outbreaks in the future.

### Transmission

In 1998, there was an unprecedented concurrent outbreak of an infectious disease in pigs with respiratory illness and humans with neurological disease in Kampung Sungai Nipah, Malaysia. A common causative agent was strongly suspected, given that the majority of human cases had direct contact with affected pigs. The disease rapidly spread as infected pigs were transported through Malaysia and into Singapore, resulting in 276 human cases, 106 fatalities, and the culling of over 1,000,000 pigs. The causative agent, named Nipah virus, was isolated from the cerebrospinal fluid of a human fatality and proved to be closely related to the Hendra virus. Despite no further cases of Nipah virus infection being identified in Malaysia, significant outbreaks occurred in India in 2001 and 2007, with multiple outbreaks and isolated cases reported in Bangladesh since 2001. Additionally, in 2014, a small outbreak of encephalitis in two villages in the Philippines was directly linked to the slaughtering and consumption of horses with neurological disease. Serologic evidence strongly indicates that horses and humans were infected with Nipah virus or a Nipah-like virus. Much like the Hendra virus, the Nipah virus originates from *Pteropus spp.* fruit bats. Zoonotic transmission of the Hendra virus in Australia and the Nipah virus in Malaysia and the Philippines occurred through an amplifying host, such as pigs and horses. In Bangladesh, zoonotic transmission is believed to occur directly from bats to humans, mainly through ingesting sap of raw dates from palm trees contaminated with the Nipah virus by fruit bats. The Henipavirus genus includes five species: Hendra virus, Nipah virus, Cedar virus, Ghanaian bat virus, and Mojiang virus. Cedar virus does not cause illness in animals or humans. Ghanaian bat virus and Mojiang virus have no direct evidence of causing disease in humans. However, Mojiang virus RNA was found in rats where workers may have acquired fatal pneumonia earlier. Henipavirus infection has been found in pigs and humans in Africa, indicating potential zoonotic transmission [13]. 

There has been a debate over the transmission route of the Nipah Virus, although being a bat-borne virus, one can say that the common transmission route is man-to-man transmission. A study done by Nikolay et al. [14] from the study in Bangladesh supports the dominance of finding transmission from person to person. However, studies from Malaysia and Singapore suggest probable transmission of virus is through workers working in pig farms suggestive of pigs being one of the cause of transmission to humans [15, 16]. 

A study conducted to observe the transmission of a virus through a hamster model indicates the importance of nasal and oropharyngeal shedding in the transmission process. The findings were consistent with previous experiments involving Nipah virus infections in pigs, where virus excretion was observed in both inoculated and contact pigs. It concluded that transmission mode from pig-to-pig, pig-to-human, and human-to-human is the same and is facilitated by direct contact with Nipah virus-containing nasal and oropharyngeal secretions. However, the specific mechanism of transmission is yet for investigation [17]. 

Transmission happens through contact with excretions or secretions of infected animals, ingestion of fruit contaminated with NiV or close contact with infected human bodily fluids [10]. 

*Malaysia*: The spread of the virus from bats to pigs is a result of consuming fruits that have been tainted by bats carrying NiV. The virus is then transmitted to humans from infected pigs through direct contact. Human-to-human transfer can occur through direct contact, air, or contaminated objects [10]. 

*Bangladesh*: It has been reported that the primary mode of transmission in Bangladesh are date palm sap consumption and person-to-person contact [9]. 

*India*: The NiV outbreaks in Bangladesh and in parts of India like West Bengal and Kerala exhibit comparable patterns in the spread of the virus among humans, characterized by direct human-to-human spread without involvement of intermediary hosts. These outbreaks primarily occurred within healthcare settings and notably impacted caregivers, and individuals in close proximity to the infected individuals [10]. 

**Fig. 2:**
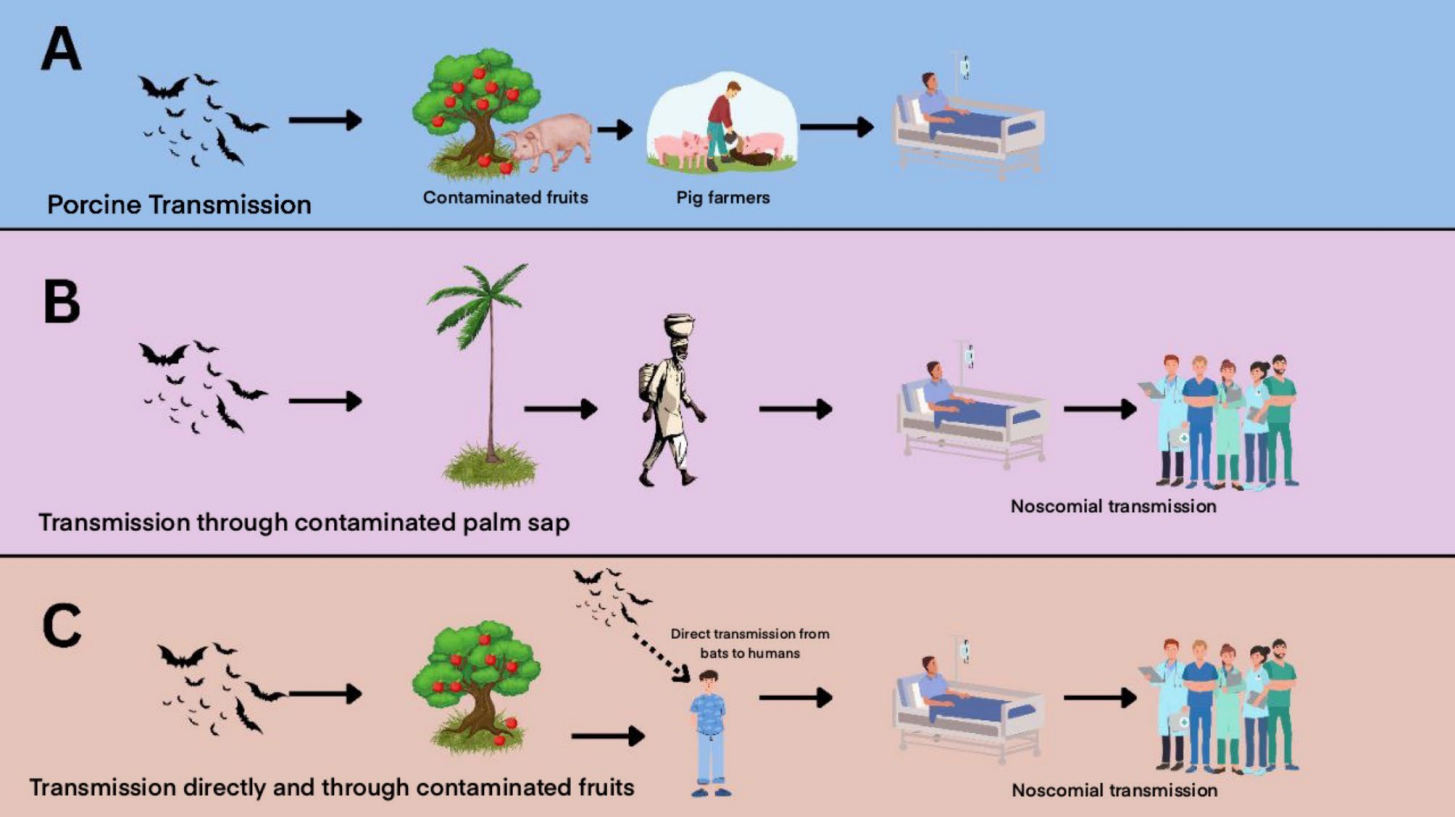
Routes of transmission of NiV in **A**- Malaysia, **B**- Bangladesh and **C**- India

Figure 2 depicts : A - In Malaysia, transmission predominantly occurs through the consumption of bat-bitten fruits contaminated with NiV-M by pigs, subsequently leading to human infections among workers handling these animals; B - Similarly, in Bangladesh, NiV-B spreads through the consumption of palm sap tainted with bat saliva and excreta, further disseminated via nosocomial transmission; C - In India, while the potential for direct bat-to-human transmission, particularly in Kerala, has been hypothesised, conclusive evidence remains insufficient. However, nosocomial transmission of NiV-B has been documented in Kerala and West Bengal, underlining the multifaceted nature of NiV dissemination across regions.

## Outbreaks

**Table 1:** Worldwide recorded cases of Nipah virus (NiV) and outbreaks, categorized by country, as of November 2023

Country	Confirmed cases	Deaths	Case Fatality Rate	Reference
Malaysia, Singapore, Philippines	293	115	34	Caruso et al., 2023[18]
India	95	69	73	
Bangladesh	335	235	65	
Total (as of 2023)	723	412	58	

### Malaysia

In 1998, near the city of Ipoh in Perak, Malaysia, the first case of NiV infection was reported among pig rearers [18]. Between 1998 and 99, three outbreaks occurred in Malaysia [10](Table 1). In these outbreaks, close contact with pigs or pig excreta was shown to be a risk factor. The infected animals themselves showed mild respiratory illness. To contain the outbreak, more than a million pigs were culled, followed by their disposal through deep burial and decontamination using quicklime. These measures, combined with other control strategies, effectively managed the situation. There is no documented evidence of transmission occurring among humans during these outbreaks. Eventually, it was discovered that Pteropus bats served as the reservoir for the infection in Malaysia. These bats transmitted the infection to pigs, acting as amplifying hosts, through the consumption of fruits bitten by the bats [9, 10]. 

### Bangladesh

In April 2001, the initial NiV outbreak, involving 13 confirmed cases(see Table 2 for definition) and resulting in 9 fatalities was documented in Meherpur, Bangladesh. Subsequently, from April 2001 to February 2015, multiple NiV outbreaks occurred yearly across different regions of Bangladesh like Naogoan, Rajbari, Faridpur, Tangail, Thakurgaon, Kushtia, Pabna, Natore, Manikganj, Gaibandha, Rangpur, Nilphamari, Madaripur, Gopalganj, Lalmohirhat, Dinajpur, Comilla, Joypurhat, Rajshahi, Jhenaidah, Mymensingh, Ponchoghor and Magura (Table 1), with certain districts experiencing repeated outbreaks of the virus [19]. In Bangladesh, the primary modes of transmission of the disease have been identified as the consumption of date palm sap and direct person-to-person contact. The outbreaks typically align with the period of sap collection, occurring from December to May [9]. Limited surveillance and healthcare resources contributed to higher mortality rates [10]. As of 2023, there were about 335 confirmed cases, resulting in 235 deaths, leading to a case fatality rate of 65% [19](Table 1).

### India

Breaking down the data from Table 1 for India, the initial Nipah virus outbreak in India took place in Siliguri, West Bengal, in 2001.With a mortality rate of 68% and 66 confirmed cases(see Table 2 for definition), this severe epidemic resulted in 45 fatalities [19]. It was followed by a smaller outbreak in 2007 in Nadia district, West Bengal, with case fatality rate of 100% where the 5 affected people died within 10 days of infection. These occurrences were near the Nipah belt in Bangladesh [9, 19]. In 2018, a separate outbreak emerged in the Kozhikode district in northern Kerala, significantly distant from West Bengal [7]. The outbreak commenced on May 2, 2018, when a 27-year-old man was hospitalized due to fever and myalgia [10]. The transmission was observed to be confined to healthcare settings, with 22 individuals getting infected from the initial patient. With a case fatality rate of 91%, 21 succumbed to the infection among the 23 confirmed NiV cases(see Table 2 for definition) [10, 19–21]. In subsequent years, isolated cases of Nipah virus infection were identified in Kerala in 2019 and 2021. The most recent outbreak occurred in 2023, involving six cases and resulting in two deaths, with a case fatality rate of 34% [19]. Between September 12 and 15, 2023, the State Government of Kerala reported six confirmed cases(see Table 2 for definition) of Nipah virus infection, with two fatalities, all occurring in the Kozhikode district. The affected individuals, aged 9 to 45 years, were exclusively males. The initial case, presenting with pneumonia and acute respiratory distress syndrome, was admitted to a hospital in late August 2023, succumbing to the illness a few days later. The subsequent five cases were close contacts, including family members and individuals at the hospital where the first case was treated; one of them died from pneumonia symptoms. By September 27, 2023, the four remaining cases were reported to be clinically stable [22]. 

### Case definitions

National Centre for Disease Control (NCDC), India has issued guidelines on the definitions of a Suspected, Probable and Confirmed case of NiV infection(See Table 2) which have been used effectively to control known outbreaks in India [23]. 

**Table 2:** Case definitions by NCDC, India [23]

Case Definitions	
Suspect Case	Individuals living in a community affected by a verified Nipah virus (NiV) outbreak and if they exhibit: • Fever with newly developed changes in mental status or seizures AND/OR • Fever with headaches AND/OR • Fever with cough or shortness of breath AND/OR • Direct exposure to a confirmed case.
Probable Case	Any individual under suspicion must meet the following criteria: • Residing in the same village/ward as the suspect/confirmed case of Nipah during the outbreak period and having passed away before complete diagnostic specimens could be collectedOR • Having direct contact with confirmed patient(s) in a hospital setting during the outbreak period and/or passing away before complete diagnostic specimens could be collected.
Confirmed Case	A presumptive case with • Detection of Nipah virus RNA through PCR in respiratory secretions, urine, or cerebrospinal fluid, OR • The isolation of Nipah virus from respiratory secretions, urine, or cerebrospinal fluid.

## Recent advances in outbreak investigation

Since the inception of infectious disease epidemiology over a century ago, mathematical representation and analysis of infectious diseases has been crucial.

Recent advancements in computers, electronic data management, internet-based data sharing and storage, rapid diagnostic testing, and genetic sequence analysis have led to widespread electronic monitoring of infectious diseases. This has also led to the increased use of mathematical models in developing and testing scientific theories and creating effective disease prevention measures.

Mathematics can aid in generating hypotheses, defining data collection techniques, and estimating sample sizes, allowing for the distinction between competing hypotheses even without reliable data [24]. 

### Classical model

As per researches the classical NiV model which is based on SIRD model developed by Kermack and McKendrick ( 1927) to investigate the spread of the disease. This is also called the classical NiV Model for predicting the Nipah Virus outbreak [24]. 

**Formula**-.$$N\left( t \right) = S\left( t \right) + I\left( t \right) + R\left( t \right)$$

Here, N(t) represent the total human population at time t, which can be divided into three distinct groups: the susceptible individuals, denoted as S(t) (healthy people who may contract the disease in the near future); the confirmed infected cases, denoted as I(t) (people who have been infected and can spread the infection to susceptible individuals); and the recovered individuals, denoted as R(t) (individuals who have recovered from the disease and either entered into immunity or have passed away) [25]. 

### The NiV integer model

This model addresses V(t) representing the amount of virus released by the infectious flying fox population into the environment. The flying fox population includes susceptible individuals (FS) and infectious flying foxes (FI), responsible for transmitting the infection to humans. The human population is divided into four subgroups: susceptible individuals (HS), infectious individuals (HI), recovered individuals (HR), and deceased individuals (HD) who died due to NiV. The shedding rate of infectious flying foxes is denoted by the parameter p, while the decay rate of the virus is represented by σ.

The NiV model is formulated using a classical differential system with integer-order derivatives. Infection transmission occurs through two modes: food-borne and human-to-human contact. Therefore, we establish the following differential equation to describe the dynamics of viral concentration [26]. 

### Basic fractional and fractal fractional theory

Fractional and fractional-finite differential operators have been shown to play a crucial role in mathematical modelling across various fields, including science, engineering, and epidemiology [27]. 

The fractional epidemic model uses derivatives with non-integer orders to capture the complex dynamics observed in certain epidemics. Fractional epidemic modelling is a more effective approach for analyzing the complex biological models with memory and hereditary effects. Therefore, this section briefly presents the fractional extension of the classic NiV transmission model [26]. 

The model includes two modes of transmission. The food-borne transmission is when the virus transmits through contaminated food and the direct person-to-person transmission from both deceased and infected humans. The model consists of seven differential equations that describe the dynamic aspects of different populations. The virus shedding by infected flying at time *t* for foxes is denoted by *V*(*t*). The population of flying foxes is divided into two subgroups: susceptible flying foxes denoted by *S*_*f*_ and infected flying foxes denoted by *I*_*f*_. The infected flying foxes *I*_*f*_ transmit the virus to the human population. The flying foxes are believed to be the natural hosts of the NiV virus.

The human population is subdivided into four subgroups: susceptible *S*, infectious *I*, recovered *R*, and deceased *D* humans, respectively. The details of each case are as follows:

Susceptible Population (S): This refers to the population that has not been infected with NiV.

Infected Population (I): This refers to the population currently infected with NiV.

Hospitalized Population (H): This refers to infected individuals who are hospitalized or under treatment.

Recovered Population (R): This refers to the population that has recovered from NiV and has developed immunity against it.

Deceased Population (D): This refers to the population that has succumbed due to NiV infection [27].

## Clinical features

The World Health Organization (WHO) indicates an incubation period of 4 to 14 days, although there have been reports of an extended incubation period lasting up to 45 days [28]. While analysing incubation periods, the incubation period varied between 4 days and 2 months, in the Malaysian outbreak, while it was around 10 days in Bangladesh & 6 to 14 days(median 9.5 days) in Kerala [10, 20].

It primarily leads to progressive encephalitis and respiratory illness, demonstrating a notable mortality rate. Nevertheless, certain individuals may remain asymptomatic. Prodromal symptoms include fever, headache, dizziness, vomiting; followed by a swift onset of progressive encephalitis leading to drowsiness, disorientation, confusion, and coma. Among central nervous system (CNS) symptoms, commonly observed are decreased levels of consciousness, brainstem dysfunction, myoclonus, areflexia, hypotonia, and cerebellar signs. Respiratory symptoms may encompass difficulties in respiration, and in some instances, a presentation similar to Acute Respiratory Distress Syndrome (ARDS) [6, 7, 29–32]. 

There are discernible variations in clinical features observed in the Malaysian and Indian outbreaks. India and Bangladesh report a higher mortality rate (70%) compared to Malaysia (40%). Respiratory illness is prevalent in 70% of patients in India and Bangladesh [11], whereas Malaysia shows no significant respiratory involvement [15]. The summary of various clinical symptoms of Nipah infection is depicted in Table 3. 

**Table 3:** Summary of clinical symptoms and signs seen in Nipah infection

Prodromal	Neurological	Respiratory
Fever	Decreased consciousness	Difficulty in breathing
Headache	Brain stem dysfunction	Acute Respiratory Distress like presentation
Drowsiness	Myoclonus	
Disorientation	Areflexia	
Confusion	Hypotonia	
Coma	Cerebellar signs	

## Diagnosis

For the diagnosis and isolation of NiV, specimens from kidney, lungs and spleen of the deceased animals are being used. Human specimens, such as nasal/throat swabs, urine, blood, and cerebrospinal fluid are collected for diagnostic purposes [10]. 

For direct detection:

1. PCR is the preferred method due to its high sensitivity, specificity, and rapidity, targeting the N (nucleocapsid) gene [9].
2. Immunohistochemistry can use formalin-fixed tissue for analysis [9, 33].
3. Virus isolation is performed in a BSL (Bio-safety level) 4 laboratory, using the Vero cell line. Cytopathic effects can be observed within 3 days [9, 10].

  Antigen detection methods include:

1. ELISA is a dependable screening tool with high sensitivity and provides rapid results. It is capable of detecting IgM and IgG antibodies against NiV. IgM antibodies can be detected in 50% of patients as early as day 1 of illness, while IgG positivity typically appears after day 18 and can persist for several months [9].
2. Serum neutralization tests are considered the gold standard but are limited to BSL4 labs. Positive sera prevent cytopathic effects, and the tests can be read at 3 days. A modified test with a 24-hour readout has been developed. In this test, the virus-serum mixture is removed after adsorption, and immunostaining is used for virus detection. Surrogate neutralization tests can be conducted using pseudo-typed viruses, which are enveloped viruses with foreign envelope proteins. These viruses can be safely handled in BSL-2 laboratories but contain NiV envelope proteins that can be neutralized by positive sera [9, 33].

## Treatment

As there are currently no approved vaccines or medications to treat NiV infection, patients can only receive supportive and preventative care. This includes maintaining prophylaxis of venous thrombosis, ensuring proper fluid and electrolyte balance, keeping airways clear, and providing mechanical ventilation when necessary [30]. 

Currently, only few pharmacological agents have shown to reduce morbidity and mortality of Nipah virus infection, and it continues to pose a difficult threat to deal with each time an outbreak occurs as based on research up to date, a handful of drugs like ribavirin, chloroquine, remdesivir, and favipiravir were experimentally proven effective on either African green monkeys or Syrian hamsters [34, 35]. Other therapeutic options under trial include Defective interfering particles (DIs) and monoclonal antibodies [34]. 

After combining the statistical protein structures and the existing papers on computational drug development, it was learned that most of the current drug development focuses on glycoprotein and fusion protein of the virus. Four other proteins can also be critical in unlocking future treatment options. They include NiV-P, NiV-M, NiV-N and NiV-L proteins and further research can potentially focus on drug development targeting these [34]. 

Tropism of Nipah and related viruses like Hendra virus for vasculature has been identified and research into their affinity for human Pluripotent Stem Cell (hPSC) derived arteries may lead to discovery of countermeasures targeting the biological pathways involved.

## Prevention strategies

### The One Health Concept

According to the WHO website, the One Health concept is an integrated approach that aims to balance and optimize the health of people, animals, and the environment. This approach brings together multiple sectors, disciplines, and communities at different levels of society to collaborate in order to prevent, predict, detect, and respond to global health threats like pandemics. One Health involves public health, veterinary health, and environmental approaches, and can be implemented to enhance the surveillance of this pathogen and reduce the risk of large NiV outbreaks.

Due to the interdependence between humans and animals, activities such as wild animal trade, hunting, deforestation, climate change, intensive agriculture, and urbanization increase human-animal contact and the risk of viral emergence. For example, in Vietnam, the possibility of the emergence of novel viruses with zoonotic potential in bats has been described, due to close human–animal contacts. Surveillance is key for preparedness against new viral emergence, for which it is necessary to conduct risk assessments, monitor wildlife or farms (pigs, poultry, etc.), and link all these data to human surveillance data. This approach could be useful to set up appropriate containment measures as quickly as possible.

In Asia, animal markets are well-known hotspots for viral emergences and, in particular, the bats that are the reservoir for NiV are sold in many street markets. The analysis of NiV genome in bats using PCR and seroprevalence studies can therefore be useful to follow the circulation of strains, including new ones, and to better understand how human outbreaks begin. Indeed, four factors must be present to initiate an epidemic: the transmission intensity in bats, the dynamic of transmission, the shedding of the virus, and the contact between bats and humans via food consumption. Surveillance of all activities that bring animal reservoirs and humans in contact should be implemented in all at-risk countries for NiV, but in the field, there are multiple organizational, financial, logistical, human, and technical challenges. The successful strategy implemented in Vietnam, based on upstream preparedness in the sanitary surveillance of wildlife, domestic animals, and humans, should serve as an example and favor the identification of the areas with the highest risk of emergence [36]. 

### Vaccines

Developing an effective vaccine against the virus would be the most definitive preventive measure. With the increasing knowledge of NiV’s molecular biology, various vaccines have been extensively researched. Early studies revealed that vaccinia virus recombinants expressing NiV-G or F proteins generated neutralizing antibodies in rodents, shielding them from fatal infections. Furthermore, a subunit vaccine containing the G glycoprotein of HeV, which is highly similar to the NiV G protein, demonstrated promising efficacy in protecting ferrets from NiV infection after exposure to lethal doses of the virus [37]. 

Research delved into a recombinant vesicular stomatitis (rVSV) vector vaccine designed to combat Nipah virus (NiV) disease using the African green monkey (AGM) model. The central element suggested for safeguarding against potential NiV infections is the development of neutralizing antibodies to NiV [38]. 

Table 4 depicts the strategies for the prevention and control of the disease formulated following the aftermath of the outbreak in Kerala in 2018 [39].

**Table 4:** Summary of Plan of Action and Future considerations for Nipah Virus

Components	Lead Agencies	What worked in Kerala	Future Considerations
**I: Institutional Framework**,** Planning and Coordination**	MoHFW, DGHS (Centre), NICD, SG, District authorities	Rapid response and mobilization of manpower and materials along with political commitment with guidelines drawn and support from the WHO	Develop national framework suited to local context incorporating lessons learnt from previous outbreaks.
**II: Surveillance and Laboratory support**	DGHS, ICMR, NICD, Integrated Disease Surveillance Project, SG	Defining the disease to identify and perform surveillance of active and recovering cases along with targeted interventions among high-risk groups. Surveillance was also done among animals.	Multiplex Polymerase Chain Revolution and Pathological autopsy for rapid diagnosis should be implemented along with international collaboration and promotion of One Health
**III: Logistics**	MoHFW, SG	Inventory assessment and DGI approval of Ribavirin. Procurement of monoclonal antibodies.	Research regarding candidate vaccines and drugs. Improvement in logistical support for field operations and safety of response teams.
**IV: Hospital Systems**	MoHFW, DGHS, SG	Protocols implemented in both private and public sector for surveillance and capacity building for handling additional surge of patients.	Effective triage facilities in the casualty and accreditation of Health care facilities. Clinical research should respect rights of patients to avoid ethical issues.
**V: Communication**		Regularly updating the WHO and National partners and providing reinforced key instructions to health-care functionaries and communication with public through mass media.	Centralized system for dissemination of key messages to both healthcare workers and public.
**VI: Regulatory Framework**	MoHFW, WHO, SG	Social distancing and Personal Protective Equipment usage	Establishing regulatory framework and provision of psychosocial support for families of patients and Healthcare providers
**VII: Community Participation**	Local bodies, NGOs and Religious organizations	Representation from different sectors towards educating and alleviating panic regarding the virus	

Abbreviations used: WHO: World Health Organization MoHFW: Ministry of Health and Family Welfare, DGHS: Director General of Health Services, NICD: National Institute for Communicable Diseases, SG: State Government, ICMR: Indian Council of Medical Research, NGO: Non-Governmental Organization

## Conclusion

The Nipah virus (NiV) is a perilous disease that presents a significant threat to global health security and is considered one of the critical zoonotic diseases. The virus is prevalent in countries located in Southeast Asia where the Pteropus fruit bats, the vector for the virus, are found. The disease has a high fatality rate and affects the brain or lungs, causing a wide variety of symptoms ranging from acute respiratory distress syndrome to encephalitis. However, diagnostic procedures are only available for BSL-3 and BSL-4 labs, and the gold standard tests are restricted to BSL-4, limiting its detection rate.

Despite the virus being discovered years ago, there remains limited knowledge about its treatment beyond conservative and symptomatic management. Several drugs and vaccines are still undergoing clinical trials, and the only promising strategy involves prevention. It is crucial to conduct further research and training on the virus to minimize the likelihood of future outbreaks and effectively manage their aftermath.

### Limitations and future prospects

**Table 5:** Ongoing clinical trials in the last five years (as of September 2024) [40].

Sr. no.	NCT Number	Study Title	Interventions	Phases	Start Date
01	NCT04199169	Safety and Immunogenicity of a Nipah Virus Vaccine	BIOLOGICAL: HeV-sG-V|BIOLOGICAL: Normal Saline Placebo	PHASE1	18-02-2020
02	NCT06221813	Study to Evaluate Safety and Immunogenicity of a Prime-Boost Regimen of rVSV-Nipah Virus Vaccine Candidate PHV02 in Healthy Adult Subjects	BIOLOGICAL: PHV02|BIOLOGICAL: Lactated Ringer’s	PHASE1	26-01-2024
03	NCT05398796	Dose Escalation, Open-Label Clinical Trial to Evaluate Safety, Tolerability and Immunogenicity of a Nipah Virus (NiV) mRNA Vaccine, mRNA-1215, in Healthy Adults	BIOLOGICAL: mRNA − 1215	PHASE1	11-07-2022
04	NCT05178901	A Phase 1 Study to Evaluate Safety & Immunogenicity of rVSV-Nipah Virus Vaccine Candidate PHV02 in Healthy Adult Subjects	BIOLOGICAL: PHV02|OTHER: Placebo	PHASE1	10-01-2022

Research focusing on the future trajectory of the Nipah virus is notably sparse, particularly when considering the critical context of ongoing vaccine development and the clinical trials currently being undertaken (see Table 5). As these trials progress, the scientific community is keenly awaiting final results that are vital for shaping effective public health strategies and interventions. The need for additional trials is underscored by the alarming statistics surrounding Nipah virus outbreaks, which have historically resulted in high mortality rates and have claimed many lives.

Current pharmacological interventions have demonstrated limited efficacy against the virus, highlighting a significant gap in our therapeutic arsenal. This is particularly concerning given the zoonotic nature of the Nipah virus, which can be transmitted from animals to humans, creating potential for widespread outbreaks. The lack of an effective vaccine compounds this issue, as many vulnerable populations remain unprotected. The absence of immunization strategies further exacerbates the risk, particularly in regions where the virus is endemic or where outbreaks have previously occurred.

Moreover, the unpredictable nature of Nipah virus outbreaks, coupled with their potential to cause severe encephalitis and respiratory distress, necessitates a multifaceted approach to research and public health planning. A comprehensive evaluation of vaccine candidates is essential, not only to determine their efficacy and safety but also to assess their potential for deployment in real-world scenarios. In addition, investigations into alternative therapeutic strategies, including monoclonal antibodies and antiviral agents, are critical for developing effective treatments.

In light of these considerations, there is an urgent need for increased funding and collaboration among researchers, healthcare professionals, and policymakers. Multidisciplinary approaches that incorporate virology, immunology, epidemiology, and clinical research will be essential in advancing our understanding of Nipah virus dynamics and in facilitating the development of robust preventive and therapeutic measures. Ultimately, a concerted effort to enhance our preparedness and response to Nipah virus and related pathogens is imperative to safeguard public health and prevent future outbreaks.

## Data Availability

No datasets were generated or analysed during the current study.
